# Questionnaire about the Adverse Events and Side Effects Following Botulinum Toxin A Treatment in Patients with Cerebral Palsy

**DOI:** 10.3390/toxins7114645

**Published:** 2015-11-06

**Authors:** Izabela Blaszczyk, Nazli Poorsafar Foumani, Christina Ljungberg, Mikael Wiberg

**Affiliations:** Department of Hand and Plastic Surgery, University Hospital of Northern Sweden, 90 185 Umea, Sweden; E-Mails: nazlifoumani@gmail.com (N.P.F.); christina.ljungberg@umu.se (C.L.); mikael.pj.wiberg@umu.se (M.W.)

**Keywords:** botulinum toxin type A, cerebral palsy, safety, adverse events, muscle spasticity

## Abstract

Botulinum toxin A (BoNT-A) injections for treatment of spasticity in patients with cerebral palsy (CP) have been used for about two decades. The treatment is considered safe but a low frequency of adverse events (AE) has been reported. A good method to report AEs is necessary to verify the safety of the treatment. We decided to use an active surveillance of treatment-induced harm using a questionnaire we created. We studied the incidence of reported AEs and side effects in patients with CP treated with BoNT-A. We investigated the relationship between the incidence of AEs or side effects and gender, age, weight, total dose, dose per body weight, Gross Motor Function Classification System (GMFCS) and number of treated body parts. Seventy-four patients with CP participated in our study. In 54 (51%) of 105 BoNT-A treatments performed in 45 (61%) patients, there were 95 AEs and side effects reported, out of which 50 were generalized and/or focal distant. Severe AEs occurred in three patients (4%), and their BoNT-A treatment was discontinued. Consecutive collection of the AE and side-effect incidence using our questionnaire can increase the safety of BoNT-A treatment in patients with CP.

## 1. Introduction

In recent decades, treatment with botulinum toxin A (BoNT-A) has increased in popularity for treatment of spasticity and several other clinical disorders [1,2]. BoNT-A is believed to help treat spasticity by interrupting the hyperactive spinal reflexes at the level of the neuromuscular junctions, which in consequence may increase range of motion, improve function, and even reduce pain [1,3,4]. The effect of the toxin is reversible and clinically effective against spasticity for 12–16 weeks [5]. The neurotoxin is considered to be safe with a low risk of severe complications [2,6]. Various studies has reported the incidence of side effects and adverse events (AEs) after BoNT-A injection in children with cerebral palsy (CP) [3,7,8,9,10,11]. The incidence of observed harms is low and transitional, but it has been observed more often in children treated with a higher total toxin dose [3,7].

In 2010, two papers were published concerning recommendations on using BoNT-A injections in children with CP [12,13]. In both, the authors recommend a dose modification according to the severity of GMFCS level, observed dysphagia, type of predominant movement disorder, muscle bulk size, nutritional status, and experience from previous BoNT-A injections. Heinen *et al.* [12] listed the AEs associated with BoNT-A injections and divided them into the following categories: generalized, focal distant, focal local and procedural [12]. The focal local AEs are fairly common but transient, such as bruising, local swelling and pain [13,14]. The generalized and focal distant AEs include generalized weakness, fatigue, flu-like symptoms, incontinence, and dysphagia. These AEs are rare and might result from a small amount of BoNT-A diffusing from the injection site and entering the circulation. Procedural side effects could be related to sedation, which is sometimes used to perform the treatment, e.g., early transitory respiratory complications or bruising at the injections site [6,14].

Love *et al.* [13] point out that efforts should be made to give patients and caregivers appropriate information about the potential risks of side effects and AEs. Educating caregivers about known side effects and AEs is necessary to minimize the risk of missing negative effects of the BoNT-A treatment. All patients should be monitored with a post-injection review to maximize the safety and quality of future treatments.

Due to this suggestion and our own experience of the difficulties in appropriate collection and reporting of side effects and AEs in our patients, we decided to educate our patients and their caregivers by the use of a questionnaire. We have not found any studies on active surveillance of harm by use of a questionnaire. Our study was a prospective collection of information from patients and caregivers about observed AEs, or side effects, after BoNT-A injection treatment for spasticity and dystonia treatment. Our hypothesis is that the questionnaire used in this study facilitates collection of information about side effects or AEs, which in turn can increase the safety of BoNT-A treatment in children with CP.

## 2. Patients and Methods

### 2.1. Study Design

This is a prospective study where active surveillance was performed using a questionnaire to collect data on the incidence of AEs and side effects in patients with CP who were treated for spasticity or dystonia in the upper or upper and lower extremity muscles in our clinic during the period of February 2010 to May 2011 (16 months). The treatments consisted of Onabotulinumtoxin A (Botox^®^, Allergan Inc., Westport, County Mayo, Ireland) or Abobotulinumtoxin A (Dysport^®^, Ipsen Ltd., Wrexham, UK) injections. The follow-ups were performed 1–3 months after treatment. All patients and caregivers had given informed consent. The Regional Ethical Review Board of Umea, Sweden, approved the study (Dnr 2013-386-31).

### 2.2. Participants

**Figure 1:**
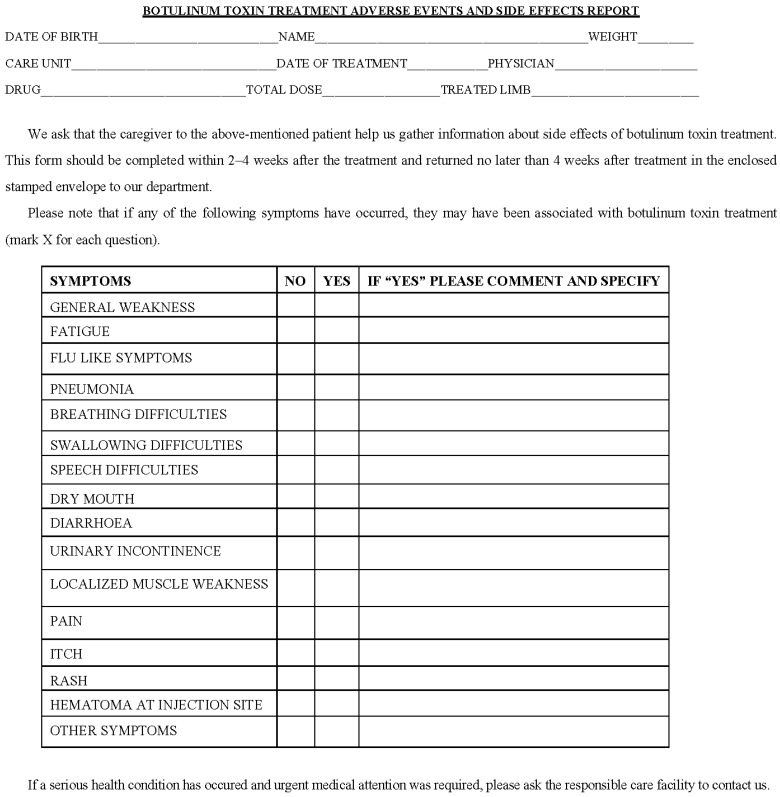
Adverse events and side effects questionnaire.

All patients (children and adults) who were treated with BoNT-A in our department from February 2010 to May 2011 were informed about the risk of AEs after BoNT-A treatment and received a questionnaire concerning AEs (Figure 1). All patients with CP were included to our study; patients with other diagnosis were excluded.

### 2.3. BoNT-A Treatment Routines

The treatment regime was based on European recommendations [12]. The number of muscles selected for BoNT-A treatment was based on the patient’s muscle disorder pattern. Injections were performed using Botox (concentration 100 U/mL) or Dysport (concentration 200 U/mL). The volume of injected toxin never exceeded 0.5 mL per injection. The injected BoNT-A dose by muscle depended on body weight, type of disorder, intensity of spasticity, or drug used. The total dose per treatment never exceeded 600 U for Botox and 1000 U for Dysport. BoNT-A injections were administrated under general anaesthesia, topic anaesthesia (EMLA), and/or conscious sedation (Midazolam). All treatments were performed using electrical stimulation as guidance. Patients were called to an occupational therapist two weeks after the treatment for physical and occupational therapy, and fitting of splints, appropriate for the functional level. One to three months after the injection treatment, the patients were called back to the investigator to evaluate the previous treatment, discuss noted AEs or side effects, and plan for the next BoNT-A injection treatment.

### 2.4. Statistical Analysis

Statistical analyses were performed using SPSS Statistics 18. The descriptive data was summarized by the mean, standard deviation, rank, categorical variable expressed as count and percentage. The incidence of AEs is presented as risk, odds ratio, 95% CI and *p* value.

### 2.5. Adverse Events Questionnaire

To capture all AEs and side effects that occurred after the BoNT-A treatment in patients with CP, a questionnaire was created which has not been validated previously. We listed the possible AEs and side effects reported in previous publications concerning use of BoNT-A injections in the children with CP [12]. Patients/caregivers were informed about the known AEs associated with BoNT-A treatment. We explained the aim of study and gave clear instruction on how to use the questionnaire. The questionnaire was designed with “yes” and “no” answers in combination with comments (Figure 1). We asked patients and/or caregivers to note if they observed any changes in status or any of the symptoms listed in the questionnaire. If a question was answered “yes”, we asked for further comments. All patients/caregivers were handed the questionnaire directly after the BoNT-A treatment had been performed. Care was taken to inform them that adverse events may be delayed and not appear until some time after the day of treatment, and therefore we asked the participants to wait a minimum of 2 weeks to fill out and send back the questionnaire. The questionnaires were sent to our department using an enclosed, pre-stamped envelope provided by us. Depending on the level of impairment, the questionnaire was filled out by the patient themselves or by a caregiver. If no posted answer was received at 4 weeks, a reminder was sent to the patient/caregiver by letter. All returned questionnaires were stored in the patient’s medical record.

All “yes”-answers, comments and missing answers were analysed and, in cases of uncertainty, were discussed at the follow-up visit, 1–3 months after treatment. All AEs and side effects were clinically verified.

### 2.6. Outcome

AEs due to systemic spread of the toxin can cause severe health consequences. Therefore, we decided to group all noted AEs and side effects found in this study into the two following subgroups:

1. Generalized (systemic) and focal distant
2. Focal local and procedural

“Other symptoms” were also classified into one of these two subgroups. Additionally, the following data was collected to perform our study: age at the time of treatment, gender, CP-type, GMFCS level, total BoNT-A dose per treatment, dose per kilogram body weight, and the number of injected muscle groups. Associations between the AE-subgroups and these variables were analysed.

For the purpose of this study patients were grouped as: minor motor impairment (GMFCS I–III) and major motor impairment (GMFCS IV–V), 1–10 and >10 years age, 10–45 kg and >45 kg body weight, total dose by treatment ≤400 U for Botox and ≤800 U for Dysport and >400 U for Botox and >800 U for Dysport, 0.1–10 U dose/body weight by treatment for Botox and 0.1–20 U dose/body weight for Dysport and >10 U dose/body weight by treatment for Botox and >20 U dose/body weight by treatment for Dysport.

## 3. Results

In total, 123 BoNT-A treatments were performed in 79 patients with CP. Only 5 patients (one treated 3 times and another one 2 times) failed to return their questionnaires. Nine patients, who were treated twice, returned the questionnaires just once, and one patient who was treated 3 times failed to return the questionnaire after one of the treatments. 105 (85%) questionnaires were returned to us from 74 patients. 89 of these were posted at 1 month post injection. 16 (15%) were received after a reminder letter. Three patients who were treated repeatedly filled out the questionnaire three times, 25 patients twice and 46 only once. Thirty-three females were treated 47 times and 41 males were treated 58 times (mean 1.4 treatments for both genders). The 74 CP patients who returned the questionnaires were aged between 1 year 6 months to 47 years 7 months (mean age 13 years 6 months, SD 7 years 8 months), weighing 10–94 kg (mean 37 kg, SD 20 kg). Eighteen patients (24%) were diagnosed with unilateral spastic CP, 38 (51%) had bilateral spastic CP, 16 (22%) had dyskinetic CP and 2 (3%) had a mixed type of CP. Twenty-eight (38%) patients had a minor motor impairment (GMFCS I–III) and 46 (62%) patients had a major motor impairment (GMFCS IV-V) (Table 1). Twenty-seven treatments (26%) were given in both upper and lower limbs; the remaining 78 (74%) treatments were in the upper limbs only. Botox was used in 86 treatments. Total dose of Botox per treatment ranged from 25–600 U, (mean 297 U, SD 162 U) and dose per kilogram body weight ranged from 1.6–21.4 U/kg, (mean 9.9 U/kg, SD 4.7 U/kg). Dysport was used in the remaining 19 treatments, with the total dose per treatment ranging from 200–1000 U (mean 629 U, SD 291 U) and the Dysport dose per kilogram body weight ranged from 2.6–22.2 U/kg (mean 11.8 U/kg, SD 5.9 U/kg).

**Table 1:** Characteristics of participants.

*n*	Sex (M:F)	Age (y:mo)	Weight (kg)	CP Type	GMFCS
74	41:33	13:6 (SD 7:8)	37 (SD 20)	USCP 18	I–III 28
				BSCP 38	
				DYSK 16	IV–V 46
				MIX 2	

M: male; F: female; y: year; mo: months; CP: cerebral palsy; USCP: unilateral spastic cerebral palsy; BSCP: bilateral spastic cerebral palsy; DYSK CP: dyskinetic cerebral palsy; MIX CP: mixed type of cerebral palsy; GMFCS: Gross Motor Function Classification System.

### 3.1. Questionnaire’s Answers Quality

Thirty-three (31%) of the returned 105 questionnaires were filled in correctly, without any observed side effects. Twenty-one (20%) questionnaires were filled in incompletely. In these, a total of 53 answers were missing. Twenty-seven (50%) of the missing answers concerned two questions, speech disorders and urinary incontinence problems. None of these patients had any speech ability and all used diapers before the study was started. Six missing answers concerned local weakness, five local pain and itching, three problems with swallowing, two dry mouth, and one influenza, generalized weakness, respiratory troubles, diarrhoea and rash. We analysed and discussed missing answers with caregivers and considered them not to be AEs as they were either already existing condition in the patient and/or difficult to assess for the caregivers (see Section 4).

### 3.2. Adverse Events

In 54 treatments, a total of 95 AEs of all types were observed in 45 patients (21 females). Forty-five AEs were focal local and procedural. Fifty generalized and/or focal distant AEs were observed in 33 treatments performed in 28 patients (17 females). Generalized AEs were reported 26 times and focal distant AEs were noted 24 times (Table 2).

Generalized muscle weakness and/or fatigue were the most often reported AEs, 21 times in 19 patients. Of these, 13 had GMFCS IV–V and six, GMFCS I–III. Only two out of these six ambulant patients were treated in the lower limbs.

We analysed the incidence of generalized and focal distant AEs and found significant associations with gender. In females the relative risk was found at 1.899 compared to males. We found a trend of association to total dose per treatment but this was not significant (*p* = 0.095). We did not find any association with the other variables (Table 3).

The future treatment plan changed for only eight patients due to reported AEs, which were assessed as significant. One patient was treated surgically due to the loss of BoNT-A effects, four patients had their treatments postponed and an additional two had their dose reduced at the next session. Three of the patients (all with GMFCS V), had adverse events that were considered severe and their future BoNT-A treatment was discontinued (Table 4).

**Table 2:** Incidence of adverse events (number of treatments = 105, number of patients = 74, F/M = 33:41).

Adverse Event’s Type (*n*)	*n* (%) AEs	*n* (%) Treatments	*n* (%) Patients	*n* (%) Female	*n* (%) Male
**All Adverse Events**	95 (100)	54 (51)	45 (61)	21 (64)	24 (59)
**Generalized (systemic) adverse events (26)**	50 (53)	33 (31)	28 (38)	17 (51)	11 (27)
generalized muscle weakness (18), fatigue (3), flu-like symptoms (5)					
**Focal distant adverse events (24)**					
swallowing difficulties (5),speech disorders (3), dry mouth (4), drooling (2), respiratory troubles (2), pneumonia (1), diarrhoea (1), nosebleeds (2), hot flashes (1), urinary incontinence (3)					
**Focal local adverse events (22)**	45 (47)	30 (29)	27 (37)	12 (36)	15 (37)
local muscle weakness (15), pain at the site of injection (3), itching (1), rush (1), swelling at injection site (1), cold hands (1)					
**Procedural adverse events (23)**					
bruising (19), leakage (2), no effect of treatment (2)					

AEs: adverse events.

**Table 3:** Risk for generalized and focal distant adverse events after BoNT-A treatment.

Variable	Odds Ratio	*p-*Value	95% CI	Relative Risk	95% CI
**Gender:** F/M	2.564	0.029	1.101–5.973	1.899	1.060–3.400
**Total dose (U):** ≥400/<400	2.171	0.095	0.875–5.390	1.651	0.945–2.885
**Body weight (kg):** ≥45/<45	1.662	0.285	0.654–4.223	1.432	0.725–2.831
**Number of treated body parts (*n*):** ≥6/<6	1.214	0.667	0.501–2.940	1.141	0.631–2.063
**GMFCS level:** IV–V/I–III	1.080	0.866	0.442–2.636	1.054	0.568–1.955
**Age (y):** ≥10/<10	0.975	0.952	0.424–2.242	0.982	0.554–1.741
**Dose (U/kg):** ≥10/<10	0.809	0.618	0.352–1.859	0.866	0.492–1.523

F: female; M: male; CI: confidence interval; GMFCS: Gross Motor Function Classification System.

**Table 4:** Characteristics of patients that had a change in treatment due to observed adverse events or side effects.

Patient	Gender/Age	CP Type	GMFCS Level	Total Dose (U)	Dose/Body Weight (U/kg)	Observed Adverse Events	Consequences
1	F/19 y	DYSK	II	280 Botox	4.8	Fatigue, hot flashes	Treatment delayed
2	M/10 y	MIX	V	160 Botox	6.4	Fatigue, pneumonia, flu-like symptoms	Treatment delayed, dose reduction
3	F/4 y	USCP	I	170 Botox	10	Speech disorders, local muscle weakness	Treatment delayed, dose reduction
4	F/2 y	USCP	II	45 Botox	4	Swallowing difficulties, flu-like symptoms, local muscle weakness	Treatment delayed
5	F/11 y	BSCP	V	400 Botox	13.3	Respiratory troubles, local muscle weakness	Discontinued
6	F/33 y	DYSK	V	1000 Dysport	22	Fatigue, respiratory troubles	Discontinued
7	M/25 y	DYSK	V	600 Dysport	15	Swallowing difficulties, Speech disorders	Discontinued
8	M/28 y	BSCP	V	300 Dysport	3.7	No effect of treatment	Surgery

y: year; F: female; M: male; CP: Cerebral palsy; GMFCS: Gross Motor Function Classification System; U :units; DYSK: dyskinetic cerebral palsy; MIX: mixed type of cerebral palsy; USCP: unilateral spastic cerebral palsy; BSCP: bilateral spastic cerebral palsy.

## 4. Discussion

The use of our questionnaire to capture the incidence of AEs and side effects after BoNT-A treatment for spasticity or dystonia in the patients with CP showed an incidence of AEs and side effects that was greater than expected. We believe the reason for this high number is due to our method of collecting data using prospective, active surveillance of harm. Our questionnaire about AEs helped us decide whether or not to change or discontinue further therapy. Three of the patients included (4%) had their BoNT-A treatment discontinued following observed AEs. In two of these, respiratory problems were assessed as severe. The third patient had swallowing difficulties which made a liquid diet necessary, resulting in substantial weight loss. In five other patients (7%) the occurrence of AEs changed the following treatments. No patients or caregivers declined treatment after being informed about the possible side effects; on the contrary, it was reported as appreciated and reassuring. We found it simple, inexpensive and effective to educate patients and caregivers on how to monitor AEs. We believe that the active surveillance of harm using our questionnaire may decrease the risk of missing AEs. It could be questioned whether all the harms found were objective, as we did not perform neurophysiological tests to confirm all of the noted events, such as general weakness. Another problem is that some AEs or side effects occurring after BoNT-A treatment were symptoms that already existed in the patients. For example, it can be difficult to observe the changes in swallowing ability in a patient who is fed through percutaneous gastrostomies because of previously existing dysphagia.

Generalized weakness and/or fatigue were, as in other studies, frequently reported side effects [3,15]. The authors found generalized weakness correlating with treatment in the lower limbs in ambulant patients, and this may be caused by either fatigue in the treated muscles or toxin leakage to the adjacent muscles [7,15]. However, in our study, generalized weakness is most likely caused by systemic spread of the toxin rather than a local muscle weakness. Only 2 out of 19 patients experiencing generalized weakness and/or fatigue were ambulant and treated in the lower limbs at the same time as the upper limbs. In the remaining 17 patients, caregivers judged them as weaker and more fatigued than usual.

We found that there is a significant difference in the incidence of generalized and focal distant AEs between males and females, and the risk for females was almost 2 times higher. Only one previous study by Jankovic and Schwartz (1991) on BoNT-A treatment of cervical dystonia suggested this correlation [16]. The weaknesses of this study are a small group, the lack of a control group and not having a validated questionnaire. Future studies need to address this.

The severity of the AEs depends on the patient’s primary condition [11,12]. For example, dysphagia can be considered a mild AE for one patient but severe for another. We agree with Heinen *et al.* [12] and Strobl *et al.* [11] that the treating physician should assess the indications for BoNT-A treatment and the risks of complications individually for each patient. In studies on harms after BoNT-A treatments in CP patients, authors report 1%–9% different adverse events [1,3,7,9,12], but Naumann and Jankovic (2004), in a review of 36 studies, described an overall rate of 25% for mild to moderate adverse events after BoNT-A treatment of different conditions [2]. It is difficult to compare our results with these studies due to the different methods of monitoring. A total of 61% of our patients reported AEs or sides effect after BoNT-A treatment but the future treatment changed as a consequence for only 11% of all patients. For three of these patients (4%), the treatment was discontinued and for five (7%) it was modified.

We believe it is important to consider and discuss the risks of adverse events and therefore we recommend the use of a questionnaire to other treating physicians. We always use our questionnaire at the patient’s first BoNT-A treatment and repeat it if any AEs or sides effects are reported. The AEs are always discussed and treatment adjustments are made if needed. The questionnaire should be validated to diminish the risk of bias.

## 5. Conclusions

Consecutive collection of AEs incidence should be routine in daily care after BoNT-A treatment. A patient/caregiver questionnaire seems to be a good tool to capture most AEs, but it needs to be validated.

## References

[1] Goldstein E.M. (2006). Safety of high-dose botulinum toxin type A therapy for the treatment of pediatric spasticity. J. Child Neurol..

[2] Naumann M., Jankovic J. (2004). Safety of botulinum toxin type A: A systematic review and meta-analysis. Curr. Med. Res. Opin..

[3] Willis A.W., Crowner B., Brunstrom J.E., Kissel A., Racette B.A. (2007). High dose botulinum toxin A for the treatment of lower extremity hypertonicity in children with cerebral palsy. Dev. Med. Child Neurol..

[4] Koman L.A., Smith B.P., Williams R., Richardson R., Naughton M., Griffin L., Evans P. (2013). Upper extremity spasticity in children with cerebral palsy: A randomized, double-blind, placebo controlled study of the short-term outcomes of treatment with botulinum A toxin. J. Hand Surg. Am..

[5] Molenaers G., van Campenhout A., Fagard K., de Cat J., Desloovere K. (2010). The use of botulinum toxin A in children with cerebral palsy, with a focus on the lower limb. J. Child. Orthop..

[6] Howell K., Selber P., Graham H.K., Reddihough D. (2007). Botulinum neurotoxin A: An unusual systemic effect. J. Paediatr. Child Health.

[7] Bakheit A.M., Severa S., Cosgrove A., Morton R., Roussounis S.H., Doderlein L., Lin J.P. (2001). Safety profile and efficacy of botulinum toxin A (Dysport) in children with muscle spasticity. Dev. Med. Child Neurol..

[8] Heinen F., Schroeder A.S., Fietzek U., Berweck S. (2006). When it comes to botulinum toxin, children and adults are not the same: Multimuscle option for children with cerebral palsy. Mov. Disord..

[9] Naidu K., Smith K., Sheedy M., Adair B., Yu X., Graham H.K. (2010). Systemic adverse events following botulinum toxin A therapy in children with cerebral palsy. Dev. Med. Child Neurol..

[10] Papavasiliou A.S., Nikaina I., Foska K., Bouros P., Mitsou G., Filiopoulos C. (2013). Safety of botulinum toxin A in children and adolescents with cerebral palsy in a pragmatic setting. Toxins.

[11] Strobl W., Theologis T., Brunner R., Kocer S., Viehweger E., Pascual-Pascual I., Placzek R. (2015). Best clinical practice in botulinum toxin treatment for children with cerebral palsy. Toxins.

[12] Heinen F., Desloovere K., Schroeder A.S., Berweck S., Borggraefe I., van Campenhout A., Andersen G.L., Aydin R., Becher J.G., Bernert G. (2010). The updated European Consensus 2009 on the use of Botulinum toxin for children with cerebral palsy. Eur. J. Paediatr. Neurol..

[13] Love S.C., Novak I., Kentish M., Desloovere K., Heinen F., Molenaers G., O’Flaherty S., Graham H.K. (2010). Botulinum toxin assessment, intervention and after-care for lower limb spasticity in children with cerebral palsy: International consensus statement. Eur. J. Neurol..

[14] Moore P., Naumann M. (2003). Handbook of Botulinum Toxin Treatment.

[15] Desloovere K., Molenaers G., Jonkers I., de Cat J., de Borre L., Nijs J., Eyssen M., Pauwels P., de Cock P. (2001). A randomized study of combined botulinum toxin type A and casting in the ambulant child with cerebral palsy using objective outcome measures. Eur. J. Neurol..

[16] Jankovic J., Schwartz K.S. (1991). Clinical correlates of resporne to botulinum toxin injections. Arch. Neurol..

